# Crescentic Glomerulonephritis Due to Enterococcal Endocarditis

**DOI:** 10.3390/jpm13081212

**Published:** 2023-07-30

**Authors:** Dragan Klarić, Marta Žutelija, Petar Šenjug, Marta Klarić, Danica Galešić Ljubanović

**Affiliations:** 1 Department of Nephrology and Dialysis, Zadar General Hospital, Bože Peričića 5, 23000 Zadar, Croatia; dragan.klaric@zd.t-com.hr (D.K.);; 2 Unit of Nephropathology and Electron Microscopy, Department of Pathology and Cytology, Dubrava University Hospital, School of Medicine, University of Zegreb, 10000 Zagreb, Croatia; dljubanov@kbd.hr; 3 Institute of Pathology, School of Medicine, University of Zagreb, Šalata 3, 10000 Zagreb, Croatia; 4 School of Medicine, University of Rijeka, Ulica Braće Branchetta 20/1, 51000 Rijeka, Croatia

**Keywords:** rapidly progressive glomerulonephritis, enterococcal endocarditis, therapy

## Abstract

Glomerulonephritis following an enterococcal endocarditis is an extremely rare and life-threatening condition. We present the case of a 71-year-old patient with rapidly progressive glomerulonephritis following enterococcal endocarditis after surgical replacement of the aortic valve. The combination of antibiotic therapy, corticosteroid therapy and haemodialysis led to an improvement in renal function; however, the severity of cardiac deterioration resulted in a fatal outcome.

## 1. Introduction

Infective endocarditis is an infection of the endocardium caused by circulating microemboli of bacteria or fungi, which affect the cardiac valves and adjacent structures and can cause serious and life-threatening conditions [1,2].

Enterococci are the third most common cause of endocarditis. Enterococcal endocarditis is a serious condition with a high mortality rate, even with prompt diagnosis and treatment [1,2]. Glomerulonephritis (GN) due to bacterial endocarditis is usually associated with blood-culture-positive bacteria, staphylococci and streptococci [1,3,4]. GN associated with infective endocarditis is usually exudative and of the proliferative histopathological type. However, crescentic or rapidly progressive glomerulonephritis (RPGN) associated with enterococcal endocarditis is extremely rare [5]. In study conducted by Boils et al., the most common biopsy finding in endocarditis-associated glomerulonephritis was necrotizing and crescentic GN (53%) and endocapillary proliferative GN (37%) [3]. In their cohort, there was no endocarditis-associated GN associated with Enterococci [3]. RPGN is defined as the presence of extensive crescents of glomeruli (over 50% glomeruli) and rapid deterioration of the renal function (usually a 50% decline in glomerular filtration rate within three months) [6,7]. Patients with RPGN usually present with nephrotic syndrome and haematuria, while the minority remain asymptomatic or have a subclinical presentation [5,6].

There are three subtypes of RPGN: pauci-immune GN, anti-glomerular basement membrane (anti-GBM) antibody-associated GN and immune-complex-mediated GN [6,8]. Enterococcal endocarditis may cause immune-complex-mediated GN associated with antigen–antibody complexes that are present in the patient’s blood. These complexes are deposited on the glomerulus and activate the inflammatory response, including complement activation, resulting in hypocomplementaemia and granular deposits of immunoglobulin in the glomerular basement membrane (GBM) [9].

We present here the case of a patient with RPGN due to enterococcal endocarditis.

## 2. Case Report

As shown in the graphic abstract, a 71-year-old female patient was hospitalized due to progressive dyspnoea in the last couple of months with swelling of the abdomen and extremities. At admission, a diastolic heart murmur parasternal left (intensity 2/6) and the systolic murmur above the aorta (intensity 2/6) were present, which correlated with aterosclerotically changed cupids of the tricuspid aortic valve with severe valve insufficiency and less severe valve stenosis on the echocardiography. Electrocardiography showed a sinus rhythm with a frequency of 70/min and left ventricle hypertrophy. The coronarography procedure showed a normal result.

Because of the echocardiography results and the clinical presentation of the patient, surgical aortic valve replacement was indicated. Cardiac surgery was performed with the assistance of an extracorporeal blood machine, and a biological valve was placed. In the postoperative course revision, resternotomy and haemostasis were performed due to enhanced drainage.

One month after the surgery, the patient was hospitalized because of acute ischemic changes in the right leg. Embolectomy of the common femoral artery and superficial femoral artery was performed, and the patient was released home in a good general condition.

Three months after the embolectomy, and four months after the valve replacement, the patient was hospitalized with a fever greater than 38.3 °C, a small reddish rash on the face and lower legs, pain under the right costal arch and in the right lumbar region and haematuria, with clinical signs of renal function deterioration (urea 11.1 mmol/L, creatinine 614 µmol/L, creatinine clearance 15.7 mL/min). As shown in Table S1, the laboratory findings revealed elevated inflammatory markers (CRP 106.5 mg/L, leukocytes 13.8 × 10^9^/L), immune thrombocytopenia (thrombocyte 22 × 10^9^/L with positive antithrombotic IgG antibodies), hypocomplementaemia (C3 0.70 g/L, C4 0.12 g/L), hypoproteinaemia (total protein 43 g/L, albumin 25.8 g/L), haemolytic anaemia (erythrocytes 2.65 × 10^12^/L, haemoglobin 82 g/L, Fe 6 µmol/L, UIBC 8 µmol/L, ferritin 571 ng/mL, transferrin 0.91 g/L, haptoglobin 0.01 g/L, LDH 487 U/L). Immunological tests showed ANCA MPO 0.4, ANCA PR3 0.2, ANA 0.1 and anti-DNA 34.

The urine culture was sterile. However, *Enterococcus faecalis* (*E. faecalis*) was isolated in three consecutive blood cultures from peripheral, venous blood. According to the antibiogram and 2015 ESC Guidelines for the Management of Infective Endocarditis, vancomycin and ceftriaxone were introduced into the therapy [10]. Since the patient was intermittently febrile for the next three days, the antibiotic regimen was changed to linezolid and teicoplanin. Doses were adjusted according to the patient’s kidney function.

At the time, chest radiography revealed bilateral pleural effusion.

Echocardiography showed a biological aortic valve (v max 2 m/s) and moderate concentric hypertrophy of the left ventricle with a good ejection fraction. The left atrium was moderately enlarged. Colour Doppler showed first-degree aortic insufficiency and second-degree mitral insufficiency. Blood pressure in the pulmonary artery was 40 mmHg. Aortal valve vegetations and aneurysm of the interatrial septum were described.

Abdominal MSCT revealed ascites around the liver and in the pelvis minor, bilateral pleural effusion and disatelectasis on the right basal side ranging up to 5 cm in size.

The control echocardiography showed an initial dilatation of up to 4.2 cm of the ascendant aorta and the stenosis of artificial valve with a peak pressure gradient (PPG)/mean pressure gradient (MPH) of 85/65 mmHg and two jets of the insufficiency. The left atrium was moderately dilated. Colour Doppler showed first-degree aortic insufficiency, third- to fourth-degree mitral insufficiency and second-degree tricuspid insufficiency.

**Figure 1:**
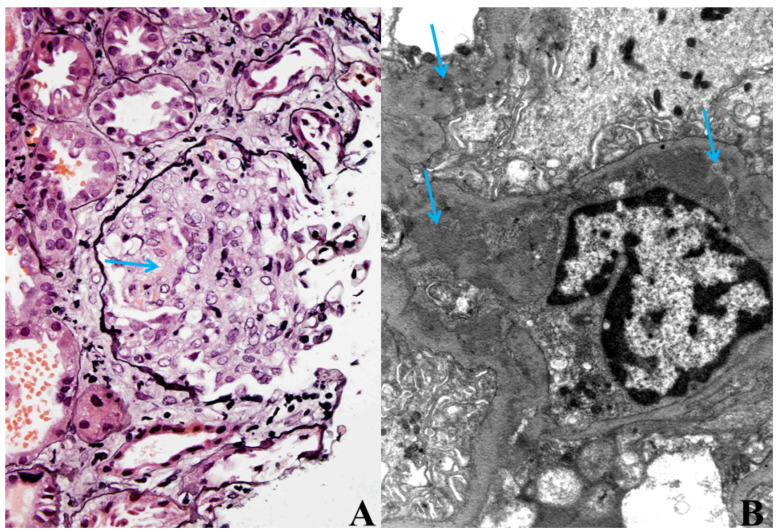
(**A**) Cellular crescent with necrosis (arrow) in glomerulus. Jones methenamine silver staining, magnification ×400. (**B**) Mesangial and subendothelial immune deposits (arrows). Electron microscopy, magnification ×10,000.

Because of the worsening of the renal function, most likely within the bacteraemia, kidney biopsy was performed. Histological analysis of the kidney biopsy showed diffuse necrotizing, crescentic, immune-mediated GN with granular deposits of IgG, IgA, IgM, C3 and C1q found predominantly in the mesangium and along the GBM. Electron microscopy confirmed this result (Figure 1A,B).

After the diagnosis of RPGN with crescents in the kidney biopsy, corticosteroid pulse therapy and haemodialysis were started, which led to renal function improvement. Intensive antibiotic treatment with linezolid and teicoplanin was also continued.

Repeated echocardiography confirmed dilatation of the ascending aorta and biological valve cupid deformation with limited mobility and separation.

Five months after the initiation of the first diagnostic procedure and treatment, the patient died due to global heart failure.

## 3. Discussion

Infective endocarditis is often associated with renal involvement including changes in the renal function and morphology. RPGN caused by *E. faecalis* is extremely rare in patients with infective endocarditis, with only few cases reported in the literature [1,5]. In our patient, bacterial enterococcal endocarditis followed by crescentic GN was diagnosed based on clinical and laboratory findings, including positive blood cultures for *E. faecalis* and the transoesophageal echocardiogram finding of biological valve cuspid degeneration and deformation with vegetations. The final diagnosis was based on histological findings of diffuse crescentic GN with extensive crescents and necrosis in the 50% of the glomeruli.

GN associated with infective endocarditis is most likely an immune-complex-mediated process associated with the presence of circulating immune complexes (CICs), hypocomplementaemia, and granular immune deposits in the GBM, although it was previously believed to be caused by emboli from the heart valves [1,5,9,11]. This is the most likely mechanism leading to the deterioration of renal function in crescentic GN caused by infective endocarditis. CICs are produced as a result of bacteraemia and cause renal damage that is immune-mediated.

GN following infective endocarditis is usually associated with streptococcal and staphylococcal infection [3]. Staphylococcal antigens have been found in glomeruli in infective endocarditis in some patients, and it is believed that they act as super antigens and increase the production of T-cell cytokines. Some other mechanisms are also speculated. However, the pathogenesis of enterococcal infection may be different [1,5,12]. Enterococcal endocarditis is most commonly caused by *E. faecalis*. *Enterococcus faecium* (*E. faecium*) usually causes bacteriemia in hospitalized patients, but *E. faecium* endocarditis is extremely rare. *E. faecalis* has an aggregation substance on its surface called gelatinase which *E. faecium* and other non-E. *faecalis* enterococci do not have. Thus, *E. faecalis* has the ability to adhere to host tissue and create a biofilm which is often antibiotic-resistant. This is believed to be a reason why *E. faecium* does not cause endocarditis even though it usually causes bacteriemia. Also, enterococci produce cytolysin with cytolytic activity. Even though all these characteristics differentiate enterococci from staphylococci and streptococci, in the end all these microorganisms are responsible for creating antigen–antibody complexes, which play a role in the development of glomerular injury [1,2]. Enterococcal endocarditis usually occurs in elderly people with a variety of underlying diseases, and it most commonly affects mitral and aortic valves [2,13]. Our patient had several comorbidities, including arterial hypertension, hypothyroidism and aortal stenosis, which led to heart insufficiency, and *E. faecalis* affected her biological valve after the surgical procedure.

The source of *E. faecalis* infection is usually the genitourinary tract, and it is induced by events including urinary infections and invasive diagnostics, as well as therapeutic procedures like cystoscopy and prostatectomy. But other invasive procedures, such as colonoscopy and liver biopsy, can also cause *E. faecalis* bacteraemia [2,14]. Our patient underwent three invasive procedures that could have created an entrance for *E. faecalis* (coronary angiography, surgical replacement of the aortic valve and an embolectomy). We hypothesize that local infection around the time of procedures (transmission from gastrointestinal tract, especially for the femoral approach) could be a source of bacteriaemia. There is hypothetical possibility of a “leaking gut”, but we did not find evidence to support that theory in our patient [15].

An alternate complement pathway is usually activated in crescentic GN caused by endocarditis. Total complement C3 and C4 levels are usually decreased. The severity of renal impairment and prognosis is associated with the degree of complement depletion [5,6,7,9,16,17].

Our patient had low C3 and C4 levels, and immunological findings showed a slightly elevated anti-DNA blood concentration, but ENA, anti-GBM and light-chain proteins in the urine and serum and serum protein immunoprecipitation were within the normal range.

It is important to emphasize that many infectious (including bacterial endocarditis) and noninfectious diseases can result in false-positive cytoplasmic antineutrophil cytoplasmic antibodies (c-ANCAs). Infectious endocarditis can be similar in clinical manifestations to ANCA-associated vasculitis such as Wegener granulomatosis or microscopic polyangiitis. But most of these infectious and noninfectious diseases with false-positive c-ANCA tests also present negative ELISA tests specific for antiproteinase 3 and antimyeloperoxidase, which is more specicfic for ANCA-associated vasculitis. It is important not to mistake infectious endocarditis with ANCA-associated vasculitis in the diagnostic process so the appropriate therapy is selected. Usually, positive blood cultures associated with skin or renal involvement, low complement levels and immune complex deposits GN help clinicians to distinguish false-positive ANCA infectious endocarditis from ANCA-associated vasculitis [18,19].

Therapeutic options and strategies for treating patients with RPGN associated with bacterial endocarditis are clearly defined. Antibiotic therapy is the basis of treatment, but additional medications and procedures are often needed, including corticosteroids, haemodialysis and plasmapheresis, and surgical removal of vegetation or the infected artificial valve [2,5].

Our patient was initially treated with vancomycin and ceftriaxone, but because of the intermittent fever, linezolid was added instead of vancomycin and further aggressive treatment with linezolid and teicoplanin was continued.

In spite of the antibiotic therapy, the patient’s renal function worsened, so corticosteroid pulse therapy was started after the kidney biopsy was performed and RPGN was diagnosed. There is uncertainty regarding the use of corticosteroids in RPGN associated with infective endocarditis because of their potential effect on infection progression. Keeping in mind that RPGN is a severe condition with a bad prognosis and knowing the benefit of corticosteroid therapy in other types of RPGN, the patient was administered corticosteroid pulse therapy. Older literature reports that in similar cases, the benefit of the use of immunosuppressive therapy outweighs the risk of worsening the infection when administrated with correctly chosen antibiotics [1,5,20]. Mantan et al. reported a case of a child with infectious endocarditis and proliferative glomerulonephritis who manifested significant proteinuria that recovered on treatment with immunosuppressants [21].

We believe that the combination of antibiotic therapy, corticosteroid therapy and haemodialysis led to the improvement in the renal function in our patient. We planned to initiate plasmapheresis therapy with the addition of albumin, but it was not necessary. Unfortunately, the severity of cardiac deterioration resulted in a fatal outcome. According to some authors, antibiotic therapy may not be sufficient, and simultaneous introduction of plasmapheresis is recommended in patients with severe hypocomplementaemia and high titres of circulating immune complexes in order to remove nephritogenic factors from the circulation [5,22,23]. On the other hand, other authors did not show any benefits from plasmapheresis in this infectious-endocarditis-associated RPGN. Ingeborg Zäuner et al. followed 39 patients with biopsy-proven RPGN in their prospective multicentre randomized study. Patients were randomized into two groups; one was treated with immunosuppressive therapy with prednisone and cyclophosphamide and the other group underwent both plasmapheresis and immunosuppression [8]. They did not find any short-term or long-term differences between the groups for the combined end points of patient death and renal failure over the 10-year observation period [8]. Similar results were presented by Cole et al., who treated 16 patients with plasmapheresis and 16 without plasmapheresis in combination with corticosteroid therapy. They did not find any significant differences in renal outcome [24].

However, some recent studies emphasize that in infective-endocarditis-associated glomerulonephritis with necrotizing lesions or crescent formation, immunosuppression does not improve renal prognosis; rather, it is associated with increased mortality [4,25].

We have presented the case of a patient who developed immune-complex-mediated RPGN because of postoperative enterococcal endocarditis. Although the patient died because of global heart failure, the combination of antibiotic and corticosteroid therapy and hemodiafiltration led to an initial improvement in renal function.

## Data Availability

Data sharing not applicable.

## Supplementary Materials

The following supporting information can be downloaded at: https://www.mdpi.com/article/10.3390/jpm13081212/s1, Table S1: Patient’s laboratory findings three months after the embolectomy, and four months after the valve replacement.
