# Supplementary material for: Crescentic Glomerulonephritis Due to Enterococcal Endocarditis
Source: J Pers Med. 2023 Jul 30;13(8):1212. doi: 10.3390/jpm13081212 (PMC10455847; doi:10.3390/jpm13081212)
Supplement: Supplementary file 1 [file jpm-13-01212-s001.zip › jpm-2526812-supplementary.pdf]

Table S1. Patient's laboratory findings three months after the embolectomy, and four months after the valve replacement.

**Laboratory finding**	**Value**	**Laboratory finding**	**Value**
erythrocytes	$2,65x10^{12}$/L	haemoglobin	82 g/L
leukocytes	$13,8x10^{9}$/L	Fe	6 µmol/L
thrombocyte	$22x10^{9}$/L	UIBC	8 µmol/L
urea	11.1 mmol/L	ferritin	571 ng/mL
creatinine	614 µmol/L	transferrin	0.91 g/L
creatinine clearance	15.7 ml/min	haptoglobin	0,01 g/L
CRP	106.5 mg/L	LDH	487 U/L
C3	0.70 g/L	ANCA MPO	0.4
C4	0.12 g/L	ANCA PR3	0.2
total protein	43 g/L	ANA	0.1
albumin	25,8 g/L	anti-DNA	34
